# A Rare Sequela of Acute Disseminated Encephalomyelitis

**DOI:** 10.1155/2014/291380

**Published:** 2014-05-26

**Authors:** Vijay Kodadhala, Saravana Devulapalli, Mohankumar Kurukumbi, Annapurni Jayam-Trouth

**Affiliations:** ^1^Department of Internal Medicine, Howard University Hospital, Washington, DC 20060, USA; ^2^Department of Neurology, Howard University Hospital, 2041 Georgia Avenue, Washington, DC 20060, USA

## Abstract

Acute disseminated encephalomyelitis is a demyelinating disease, typically occurring in children following a febrile infection or a vaccination. Primary and secondary immune responses contribute to inflammation and subsequent demyelination, but the exact pathogenesis is still unknown. Diagnosis of acute disseminated encephalomyelitis is strongly suggested by temporal relationship between an infection or an immunization and the onset of neurological symptoms. Biopsy is definitive. In general, the disease is self-limiting and the prognostic outcome is favorable with anti-inflammatory and immunosuppressive agents. Locked-in syndrome describes patients who are awake and conscious but have no means of producing limb, speech, or facial movements. Locked-in syndrome is a rare complication of acute disseminated encephalomyelitis. We present a case of incomplete locked-in syndrome occurring in a 34-year-old male secondary to acute disseminated encephalomyelitis. Our case is unique, as acute disseminated encephalomyelitis occurred in a 34-year-old which was poorly responsive to immunosuppression resulting in severe disability.

## 1. Introduction

Acute disseminated encephalomyelitis (ADEM) which is commonly preceded by an infection is an inflammatory and demyelinating disorder of central nervous system [1]. Exact pathogenesis is still not completely understood. Locked-in syndrome (LIS) is a rare neurological condition caused by an insult to the brainstem [2]. We present a case of a young man who developed an incomplete LIS as a sequel of ADEM.

## 2. Case Presentation

A 34-year-old male with past medical history of protein S deficiency presented to emergency room with a two-week history of worsening headaches and three-day history of subjective fevers, nausea, and vomiting. Over the counter medications did not relieve his symptoms. On the day of presentation, he experienced difficulty in walking and according to his friends he seemed to be mildly confused. He neither complained of any visual changes nor had any seizures. He is of Indian origin, professional, and married with a child. His history was negative for smoking, alcohol, and illicit drug use. He came to United States six months prior to onset of these symptoms for his job. He did not have any sick contacts with or exposure to wildlife. He did not receive any vaccination within the recent past.

On physical examination his vital signs are within normal limits. He was oriented to only person and place but he did not have any other focal neurological deficits. Other systems examinations were within normal limits. His initial basic labs were within normal limits. Computerized tomography (CT) scan of head did not reveal any acute intracranial process.

A spinal tap was performed and patient was started on empiric antibiotic therapy and Acyclovir. Cerebrospinal fluid (CSF) analysis was consistent with aseptic meningitis (White Blood Cell Count (WBC)-287 cells/mL with lymphocytic predominance, glucose at 30 mg/100 mL, and protein at 236 mg/100 mL) and did not reveal any albuminocytological dissociation. Based on CSF findings, antibiotics were discontinued, Acyclovir was continued, and its course was completed.

**Figure 1:**
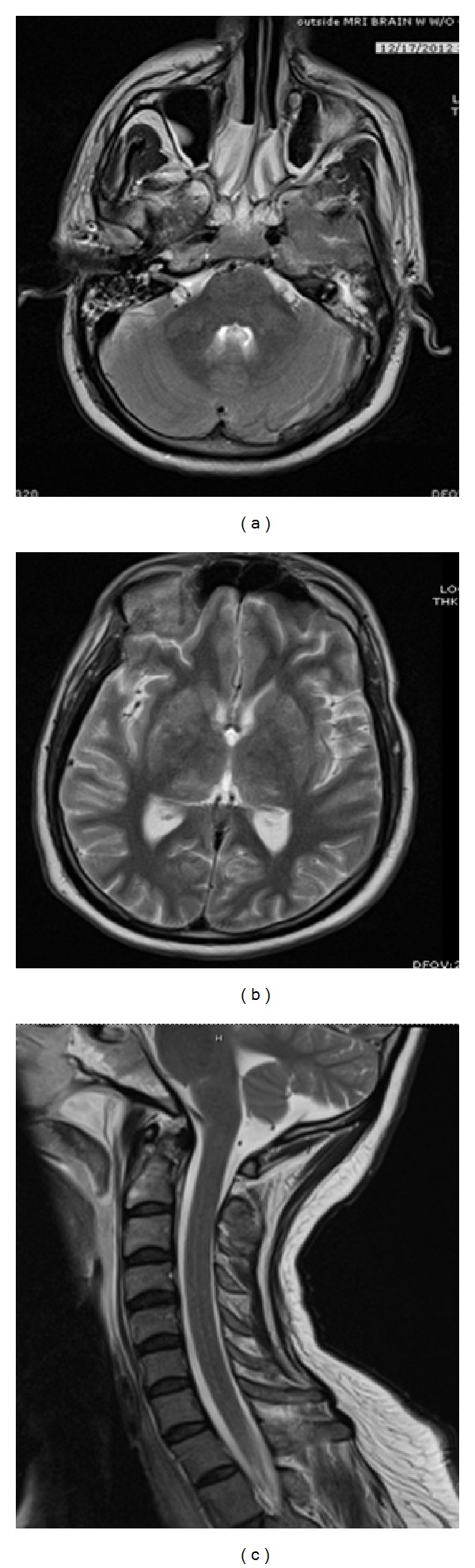
(a) MRI brain showing increased T-2 signal in middle cerebellar peduncles. (b) MRI brain showing increased T-2 signal in bilateral basal ganglia. (c) MRI cervical spine showing mild diffusely increased T-2 signal.

On the second day of admission, his mental status worsened; he developed seizure and went into respiratory failure which needed intubation. Magnetic resonance imaging (MRI) brain performed during that time course revealed symmetrical increased T-2 (Figures 1(a) and 1(b)) signal in bilateral basal ganglia, thalami, and middle cerebellar peduncles and did not show any gross enhancement on postcontrast images except for small amount of subependymal enhancement along the occipital horns. Increased FLAIR signal along the cerebral leptomeningeal surface in certain areas and along brainstem was also evident. Multiple microhemorrhages are identified in corpus callosum, gray white matter junction, and brainstem. Mild diffusion restriction in corpus callosum and left hippocampus was present. Findings were consistent with ADEM possibly infectious/inflammatory/autoimmune mediated. MRI cervical spine showed mild enlargement of cervical cord and mild diffusely increased T-2 signal. Postcontrast images were without abnormal enhancement or focal lesions. MRI of thoracic and lumbar spine was normal.

Ten days after admission, patient developed ascending lower limb weakness and ultimately went into comatose state. He was treated with steroids, IVIG, and plasmapheresis for possible parainfectious demyelinating polyradiculopathy or parainfectious encephalomyelitis. Nerve conduction and electromyography studies were not suggestive of the Guillain-Barre syndrome. Patient did not respond to immunomodulators, so the Guillain-Barre syndrome was not considered as possible cause of patient's medical condition.

Further studies from CSF analysis were positive for oligoclonal bands, West Nile IgG, and Epstein-Barr's virus (EBV) IgG antibodies and negative for West Nile IgM, HSV polymerase chain reaction (PCR), West Nile PCR, lyme titers, VDRL, anti-Hu antibodies, and acid fast bacilli. CT scan of chest, abdomen, and pelvis and ultrasound of testis did not reveal any primary malignancy. Serum anti-NMDA receptor antibodies and paraneoplastic panel were negative.

**Figure 2:**
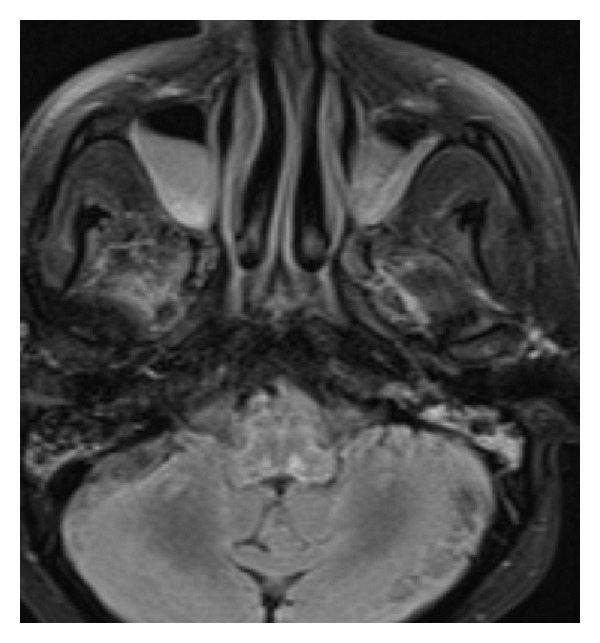
MRI brain showing increased T2 FLAIR signal in brainstem.

Three weeks after admission, patient was still unresponsive with no gag, cough, blinking to threat, eye tracking or deep tendon reflexes. Due to his persistent altered mental status, brain biopsy (right frontal approach) was performed. It did not reveal any evidence of inflammation, caseating, or noncaseating granulomas. After five weeks, MRI brain (Figure 2) was repeated which revealed more pronounced brainstem involvement and still has persistent signal abnormality along sulci in postcontrast and FLAIR images. Electroencephalogram revealed evidence of diffuse encephalopathy.

After approximately six weeks, he was alert and awake and began tracking with his eyes. He responded to simple commands like facial and neck movements (given in his native language) and did not respond to the commands given in English (Even though he knew how to speak English before the onset of illness). Unfortunately eye-coded alphabetical communication system was not available in his native language, and thus it was not performed. Pupils were equal and reactive. Extraocular movements were intact and full. There was some nystagmus noted, more on the left side than the right side. Fundus examination was normal. No facial asymmetry was noted. He has some intact sensation over the face bilaterally to pinprick. He was able to open his mouth partially. No words are being produced at this time. He was completely nonverbal.

On motor examination he was quadriplegic. His power both in upper and lower extremities was 0/5 on Medical Research Council scale. Some occasional flickers of contractions were seen in bilateral lower extremities. He had some sensations intact in the upper limbs, face, and chest area symmetrically with pinprick stimulation, but no sensation was appreciated below the waist. Deep tendon reflexes are 1+ in bilateral upper and lower extremities. Hypertonia was seen in all four extremities. Plantars are upgoing bilaterally. Because of the above findings, he was diagnosed to have incomplete LIS.

Because of prolonged respiratory failure, patient got tracheostomy and percutaneous endoscopic gastrostomy tube placement for feeding. Patient's hospital course was complicated by repeated pulmonary infections. So detailed neuropsychological testing was not performed.

Patient was discharged to nursing home. His stay at nursing home was complicated by recurrent health care associated pneumonia and urinary tract infections which needed multiple admissions to hospitals.

**Figure 3:**
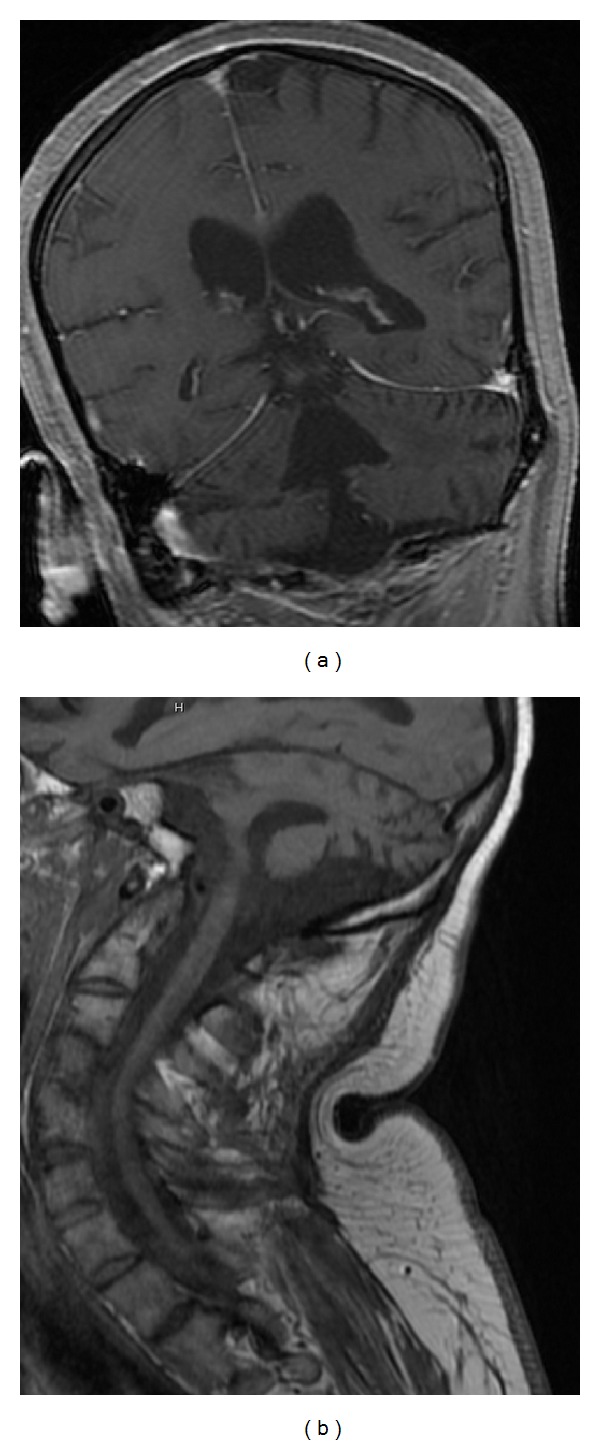
(a) and (b) (Seven months after initial MRI). MRI brain (a) showed marked atrophy of the brainstem, predominantly in the pons. MRI cervical spine (b) showed increased volume loss in the cervical cord on T-1 weighted image.

Approximately seven months later repeat MRI brain (Figure 3(a)) showed marked atrophy of the brainstem, predominantly in the pons and abnormal signal in the bilateral middle cerebellar peduncles. MRI cervical spine (Figure 3(b)) showed increased volume loss in the cervical and upper thoracic cord. His physical exam was still consistent with an incomplete LIS.

## 3. Discussion

ADEM is a demyelinating disease of the central nervous system usually preceded by a viral or bacterial infection [1]. Exact pathogenesis is still not completely understood. In a prospective study it was found that 61 percent survived without any neurological sequel, 18 percent were mildly impaired, 14 percent were severely impaired, and 1 percent remained in vegetative state [3]. LIS is a rare neurological condition caused by insult to brainstem [2]. We present a rare case of a young adult who developed incomplete LIS due to ADEM most likely secondary to viral etiology.

LIS is characterized by paralysis of the lower cranial nerves and quadriplegia. Patients retain consciousness and can communicate by vertical eye movements and eye blinking [2]. The first published case of LIS in 1947 was described in a patient with an infarction in the territory of the vertebral-basilar artery system that manifested with tetraplegia, intact consciousness, and no verbal communication [4]. Plum and Posner coined the term “locked-in” in 1966 to describe the state of anarthria with preserved consciousness and quadriplegia [5].

LIS is of 3 types: classic, incomplete, and total [6]. Classic LIS patients have anarthria and quadriplegia with preserved consciousness, blinking, and vertical eye movements. Patients with incomplete LIS additionally possess some voluntary motor movements in the neck or fingers. Patients with total LIS have no movements, including that of the eyes or eyelids [7]. Our patient was diagnosed with incomplete LIS as he manifested intact and full extraocular movements in all directions and additional voluntary motor movements like trying to open his mouth.

LIS is most frequently caused by thrombotic occlusion of the basilar artery. Less frequently, basilar artery dissection, brainstem hemorrhage, central pontine myelinolysis, and primary or secondary malignant infiltration of the basis pontis can also cause LIS [8]. Other less common causes of LIS include brainstem encephalitis, polyneuritis, myasthenia gravis, poliomyelitis, EBV, pneumococcal meningitis, and West Nile viral infection [9].

A lesion in the ventral pons that causes interruption of the corticospinal tracts bilaterally causes quadriplegia. Corticobulbar tract lesion affects cranial nerves XI and XII, which are involved in control of the facial, pharyngeal, and tongue muscles for speech and vocalization. Supranuclear pathway preservation due to the sparing of the midbrain tectum allows the patient with LIS to open eyes, produce vertical eye movements, and blink [10, 11]. However, other eye movements may be adversely affected due to lesions in the pons, including the paramedian pontine reticular formation or abducens nucleus. Sensation is preserved due to the sparing of spinothalamic tracts, which lie dorsally in pons [10, 11].

Viral encephalitis can be either primary or postinfectious. Primary infection is characterized by viral invasion of the central nervous system. Neuronal involvement can be identified on histologic examination and virus can often be cultured from brain tissue. In ADEM, which is postinfectious, a virus cannot be detected or recovered and the neurons are spared. Perivascular inflammation and demyelination are prominent in this entity. These features suggest that ADEM is an immune-mediated disease [12]. In our patient, biopsy of cerebral cortex and meninges were normal. Biopsy from brainstem which was predominantly involved later in the disease course would be definitive but is impractical to perform.

A wide variety of viruses can infect the nervous system [13]. Most viruses are capable of causing either meningitis or encephalitis. Etiological agent can vary with the season of the year and geographical location [14]. Altered mental status, altered behavior and personality changes, motor or sensory deficits, and speech or movement disorders are expected depending on the location of inflammation. Frequent causes of encephalitis secondary to infection must be excluded before concluding to an acute form of postinfectious inflammatory CNS disorder [12]. No specific viral etiology was confirmed in our patient even though West Nile IgG was elevated.

In our patient anti-NMDA receptor encephalitis was unlikely because CT scan of chest, abdomen, and pelvis and ultrasound of testis did not reveal any primary malignancy. Serum anti-NMDA receptor antibodies and paraneoplastic panel were negative. Neuromyelitis optica was not considered because of atypical radiological findings and unresponsiveness to steroids/IVIG. Also CSF analysis revealed lymphocytes but not neutrophils and eosinophils which make NMO unlikely even though aquaporin 4 antibodies were not tested. Sarcoidosis was not considered and was not investigated because our patient does not have any clinical features of sarcoidosis; patient did not respond to steroids. Brain biopsy ruled out sarcoidosis.

The location of abnormal signal in CT or MRI imaging can sometimes be suggestive of specific etiologies. Temporal lobe involvement is common in herpes encephalitis, although other herpes viruses (e.g., varicella, EBV, and human herpesvirus 6) can also produce this clinical picture [15, 16]. The basal ganglia or thalamus involvement is common in respiratory viral infection, arbovirus, Creutzfeldt-Jakob's disease, and tuberculosis [17–20]. Our patient had basal ganglia, thalamus, middle cerebellar peduncle, and brainstem involvement.

Diagnosis is based on CSF analysis, serology, culture, PCR, and brain biopsy. PCR revealed a viral cause in 36 percent of cases in a study [21]. In a study done by Whitley et al., with brain biopsy, 45 percent had the diagnosis confirmed, 9 percent had another virus detected, and 9 percent had another treatable cause [22]. Our patient was diagnosed with ADEM based on the clinical presentation, the radiological findings, and ruling out infectious etiology. There are no specific therapies for most CNS viral infections except for very few. Our patient has poor response to treatments as mentioned above and developed incomplete LIS which is very rare.

## 4. Conclusion

ADEM is an uncommon disease characterized by inflammation and demyelination of central nervous system. It appears to be an immune reaction due to the infection or vaccination and carries a good prognosis with immunosuppressive treatment. Our patient had a poor response to the aforementioned treatment and developed an incomplete LIS, which is a rare outcome.

## Abbreviations

ADEM: Acute disseminated encephalomyelitis
CSF: Cerebrospinal fluid
CT: Computed tomography
EBV: Epstein-Barr's virus
FLAIR: Fluid-attenuated inversion recovery
IgG: Immunoglobulin G
IgM: Immunoglobulin M
IVIG: IV immunoglobulin
LIS: Locked-in syndrome
MRI: Magnetic resonance imaging
PCR: Polymerase chain reaction
VDRL: Venereal Disease Research Laboratory
WBC: White blood cell.
